# The Mediating Effect of Physical Fitness on the Relationship Between Developmental Coordination Disorder and Physical Activity in School-Aged Children—An Observational Study

**DOI:** 10.3390/life16060870

**Published:** 2026-05-22

**Authors:** Huynh-Truc Tran, Wen-Chao Ho, Li-Wei Chou, Yao-Chuen Li

**Affiliations:** 1 Department of Public Health, China Medical University, Taichung 406040, Taiwan; huynhtruc2105@gmail.com (H.-T.T.); wcho@mail.cmu.edu.tw (W.-C.H.); 2 Department of Physical Therapy and Graduate Institute of Rehabilitation Science, China Medical University, Taichung 406040, Taiwan; 3 Department of Physical Medicine and Rehabilitation, China Medical University Hospital, Taichung 404332, Taiwan; 4 Department of Physical Medicine and Rehabilitation, Asia University Hospital, Asia University, Taichung 413505, Taiwan

**Keywords:** developmental coordination disorder, physical activity, accelerometry, mediation, flexibility, lower-body strength

## Abstract

Evidence remains limited on the interconnections between developmental coordination disorder (DCD), health-related physical fitness—including body composition, flexibility, strength of lower body, and cardiorespiratory fitness—and objectively measured physical activity (PA) in school-aged children. This study aimed to (1) examine differences in physical fitness and PA between children with and without DCD and (2) investigate whether physical fitness functions as a mediator in the association between DCD and PA in school-aged children. Sixty-three children aged 6.5–8 years (12 DCD, 19.05%) who provided valid data were enrolled. Mediation analysis was conducted using the PROCESS macro for SPSS. Flexibility significantly mediates the relationship of DCD to vigorous PA (VPA) (effect = 3.202, bootstrap SE = 1.682, 95% bootstrap CI = 0.463, 7.078), as well as DCD to moderate-to-vigorous (MVPA) (effect = 5.194, bootstrap SE = 2.903, 95% bootstrap CI = 0.434, 11.778). Additionally, there was a significant mediating effect of lower muscle strength on the relationship between DCD and VPA (effect = −1.943, bootstrap SE = 1.297, 95% bootstrap CI = −5.112, −0.021), and DCD and counts per minute (CPM) in axis 2 (effect = −34.388, bootstrap SE = 20.212, 95% bootstrap CI = −80.819, −1.353). The findings highlight flexibility and lower-body strength as potential mechanisms underlying the association between DCD and PA participation. These factors may represent candidate intervention targets; however, their roles require confirmation in larger samples and longitudinal designs.

## 1. Introduction

Physical activity (PA) is defined as body movement produced by muscle contraction and increased energy expenditure [1]. Engaging in adequate levels of PA offers numerous health benefits, including physiological benefits such as the prevention of overweight or obesity and a decreased risk of premature mortality, cardiovascular disease, type 2 diabetes, metabolic syndrome, and some forms of cancers, as well as psychological benefits, such as improved mental health, attention, focus, mood, self-image, autonomy, and social well-being [2]. To achieve these health benefits, school-aged children are recommended to accumulate at least 60 min of moderate-to-vigorous physical activity (MVPA) per day [3]. However, more than half of children and youth do not meet this recommendation and instead spend a large amount of time in sedentary behaviors [4].

Children with developmental coordination disorder (DCD) have been found to be at a significant risk for reduced PA due to their poor motor skills [5,6,7]. According to the Diagnostic and Statistical Manual of Mental Disorders—Fifth Edition (DSM-V), DCD is one of the most common neurodevelopmental disorders, affecting approximately 5–6% of all school-aged children [8]. Due to its nature, this disorder significantly affects both gross and fine motor skills, making it challenging for children to perform everyday tasks at home and school, and hindering the acquisition of new or complex motor skills required for PA. A recent systematic review and meta-analysis has rigorously investigated PA in children with DCD and demonstrated a significant difference in moderate-to-vigorous PA (MVPA) between children with and without DCD; specifically, school-aged children with DCD showed significantly lower levels of MVPA compared to their typically developing (TD) peers [9]. This lack of PA may subsequently lead to various physical and mental health problems, such as an increased risk of obesity-related chronic diseases and cardiovascular disease later in life [10,11], low self-perceived competence [12], reduced quality of life [13], and poor physical fitness [14]. Although an increasing number of studies have investigated PA in children with DCD [5,6,7,12,15], the causal associations between these factors and the underlying mechanisms remain unclear.

From a theoretical perspective, the relationship between motor competence, physical fitness, and PA could be understood through developmental and ecological frameworks. The developmental model of motor competence suggests that motor competence influences PA both directly and indirectly through its impact on health-related physical fitness [16]. Children with lower motor competence may be less likely to successfully and sufficiently engage in PA, which may limit opportunities to develop physical fitness components, such as muscular strength and cardiorespiratory fitness, which may further constrain sustained engagement in MVPA [16]. In addition, ecological perspectives emphasize that PA behavior is shaped by the interaction among individual constraints, task demands, and environmental factors [17]. Within this framework, DCD might be conceptualized as an individual constraint that limits children’s ability to engage in physical activities, thereby reducing participation opportunities and contributing to lower physical fitness over time. While the ecological framework explains how behavior emerges from these constraints, the role of physical fitness as an intermediary mechanism linking DCD and PA is more directly supported by developmental models. Taken together, these perspectives suggest that motor impairments associated with DCD may indirectly contribute to lower levels of PA through their impact on physical fitness.

Multiple dimensions of physical fitness, including body composition, flexibility, strength of the lower body, and cardiorespiratory fitness, may influence PA behaviors in children with DCD [18,19,20,21]. Existing studies have revealed that children with low motor proficiency or DCD tend to exhibit higher body mass index (BMI) [18], lower muscle strength [19], and poorer flexibility [20]. For instance, increased BMI may hinder children’s ability to engage in activities such as running, jumping, and flexibility due to the mechanical disadvantage of excess weight and the higher oxygen cost of locomotion [21]. Similarly, inadequate muscular strength may result in poor posture, musculoskeletal problems (e.g., lower back pain or joint laxity), and difficulty participating in sports that require force production [21]. Moreover, poor flexibility may reduce movement efficiency [21], while lower cardiorespiratory fitness limits endurance and the ability to sustain MVPA [14]. Collectively, these interrelated components of physical fitness are essential for both motor performance and sustained engagement in PA [18,19,20,21], and therefore represent important targets for early identification and intervention.

Despite growing evidence regarding physical inactivity in children with DCD, the mechanisms underlying this relationship remain unclear. In particular, limited research has examined whether health-related physical fitness (i.e., body composition, flexibility, strength of the lower body, and cardiorespiratory fitness) mediates the relationship between DCD and PA in school-aged children [18,19,20,21]. In order to enhance the effectiveness of programs and interventions targeting the improvement in PA in children with DCD [22], it is important to identify modifiable factors that could be targeted. Therefore, the objective of this study was twofold: (1) to investigate whether there are differences in the components of health-related physical fitness (i.e., body composition, flexibility, strength of lower body, and cardiorespiratory fitness) and objectively measured physical activity between children with and without DCD, and (2) to examine whether physical fitness mediates the relationship between DCD and PA. It was hypothesized that children with DCD would perform worse on physical fitness and PA, compared to children with TD, and that the association between DCD and PA would be mediated by these components of health-related physical fitness.

## 2. Materials and Methods

### 2.1. Participants

A total of 63 children aged 6.5 to 8 years participated in the study. Motor coordination was evaluated for all participants using the Movement Assessment Battery for Children–Second Edition (MABC-2). According to the MABC-2 percentile scores, 12 children (9 boys and 3 girls; mean age = 7.89 ± 0.52 years) were assigned to the DCD group (≤16th percentile), while 51 children (24 boys and 27 girls; mean age = 7.82 ± 0.62 years) were classified as typically developing (TD; >16th percentile). Diagnostic classification of the DCD group was further verified in accordance with the Diagnostic and Statistical Manual of Mental Disorders, Fifth Edition (DSM-5) criteria [8]. Detailed descriptions of all assessment procedures are provided below. Participants in both groups had no history of intellectual disability (IQ < 70) or neurological or movement-related conditions that could account for motor impairments. Caregivers were also invited to take part in the study. Written informed consent was obtained from parents and from children aged over seven years.

### 2.2. Research Design and Procedure

The present study employed a secondary analysis of data derived from the Stress, Motor Coordination, and Activity Relationships in Taiwanese children (SMART) project, a three-year longitudinal investigation approved by the Central Regional Research Ethics Committee of China Medical University (CRREC-108-159). The recruitment period started in September 2019 and ended in September 2020. A comprehensive description of the SMART study design and procedures has been published previously [23]. In brief, children were recruited from the kindergartens and followed up for two years. They were included if the chronological age was between 4 and 6 years. However, children were excluded if neurological diseases/disorders or physical disability were reported by parents or teachers. Baseline assessments included evaluations of motor coordination and nonverbal intelligence, conducted using the MABC-2 test [24] and the Test of Nonverbal Intelligence—Fourth Edition (TONI-4) [25], respectively. Furthermore, the assessments of BMI, strength of the lower body, flexibility, cardiorespiratory fitness, and self-concept were repeatedly administered by trained research assistants in a lab once a year. Following each assessment session, children were provided with accelerometers to objectively monitor PA over the subsequent seven-day period. Parents were then taught how to accurately wear the accelerometer and to record the time of day when the accelerometer was worn and removed in a diary. After data collection was completed, the accelerometer with the diary was placed into an envelope with bubble wrap and mailed to our lab. Informed consents were obtained from all participants’ parents. In this study, data collected in the second or third year were used when participants began to go to primary school.

### 2.3. Measures

#### 2.3.1. Physical Activity

PA was measured using the ActiGraph wGT3X+ accelerometer (ActiGraph, Pensacola, FL, USA). The ActiGraph wGT3X+ accelerometer was a light and small device that was fastened around the right waist of the child for seven consecutive days. Children wore the accelerometer during all waking hours and only be removed when they were sleeping or exposed to water, such as taking a shower or swimming. Parents were requested to keep a diary to record the time of day when the accelerometer was placed on their children and taken off. To ensure that the daily accelerometer data were reasonably and accurately representative, at least 600 min of wear time per day on at least 3 days were required [26,27,28]. Furthermore, in order to accurately capture PA in our participants, 3-s epochs were used for children [26]. Activity counts were summed and expressed as counts per minute (CPM)**.** Physical activity intensity was classified using the Evenson cut points [29], with thresholds of ≤100 CPM for sedentary behavior (SB), 101–2295 CPM for light physical activity (LPA), 2296–4011 CPM for moderate physical activity (MPA), and ≥4012 CPM for vigorous physical activity (VPA). Time spent in moderate-to-vigorous physical activity (MVPA) was calculated as the sum of MPA and VPA, given its established association with health outcomes in children [30]. Total physical activity (TPA) was defined as the total accumulated activity counts per day. In addition to overall activity intensity, CPM were also derived separately for each accelerometer axis to reflect movement in different planes: vertical (axis 1), anteroposterior (axis 2), and mediolateral (axis 3) directions.

#### 2.3.2. Developmental Coordination Disorder

All children were tested for motor competence using the MABC-2 test at each assessment period. The MABC-2 test is a widely accepted tool in DCD diagnosis that measures the severity of motor impairment. In the SMART study, the Age Band 1 (i.e., 3–6 years old) and 2 (i.e., 7–10 years old) of the MABC-2 test were utilized at baseline to assess motor coordination in preschool children aged between 4 and 6 years. The tool comprises eight items under three motor skill categories: manual dexterity, aiming and catching, and static and dynamic balance. It has demonstrated good reliability and validity [24]. Children scoring at or below the 16th percentile were identified as exhibiting motor difficulties, in accordance with Criterion A of the DSM-V. To address Criterion B, parents completed the MABC-2 Checklist to evaluate whether motor difficulties interfered with the child’s daily activities or learning-related tasks in preschool or school settings. When children participated in the SMART study, all of them were 4 to 6 years old, which met the UNICEF’s definition for early developmental years and Criterion C of the DSM-V [31]. To rule out intellectual impairment in accordance with Criterion D, fluid intelligence was assessed using the Chinese version of the Test of Nonverbal Intelligence–Fourth Edition (TONI-4) [25]; no child met the criterion for intellectual disability (IQ < 70). In addition, parents provided information on their child’s medical history to confirm the absence of neurological or medical conditions that could otherwise explain motor impairments.

#### 2.3.3. Health-Related Physical Fitness

There are four health-related physical fitness variables included in the current study: body composition, strength of the lower body, flexibility, and cardiorespiratory fitness. This study calculated BMI to indicate body composition, which was identified by the Centers for Disease Control and Prevention as an effective and reliable screening tool for evaluating an individual’s weight status in relation to their height. In this study, we measured children’s standing height barefoot to the nearest 0.1 cm using a stadiometer. We also recorded their body weight without shoes to the nearest 0.1 kg using an electronic scale (InBody H20, InBody Co., Ltd., Seoul, Republic of Korea). The results of both height and weight were utilized to calculate BMI (i.e., kg/m^2^).

Flexibility was measured using the Sit-and-Reach Test (SRT) with a testing equipment (arrow-type seated forward bending tester, AC696A, Accuratus, New Taipei City, Taiwan). The SRT is commonly used to measure flexibility, specifically hamstring extensibility [32]. While conducting the SRT, participants sat on the floor with bare feet and kept both knees extended. With the palms facing downwards and one hand placed on top of the other, children were asked to reach forward along the arrow-type seated forward bending tester as far as possible. One practice trial was followed by two testing trials. The distance was recorded, and the best performance was used to indicate children’s flexibility.

Strength of the lower body was measured using the Standing Long Jump Test, which was considered an inexpensive and simple test to assess lower leg power [33]. All participants were asked to stand with both feet behind a starting line on a mat with a distance scale and then encouraged to jump as far as possible. One practice and two testing trials were conducted. The trial was considered successful if participants could land on both legs and remain in a stable position. Their jumping distance was recorded in centimetres, and the best performance was used to indicate children’s strength of lower body.

The six-minute walk test (6MWT) was used to assess children’s cardiorespiratory fitness [34]. The child put a wearable device (Polar A370, © Polar Electro, Kempele, Finland) on the wrist to track the change in heart rate. Before the 6MWT, they needed to quietly sit on a chair for three minutes, and then the resting heart rate was measured. During the test, children were instructed to walk at their comfortable pace as far as they could within six minutes, and the research assistants would walk with them to record the distance using the distance measuring wheels (King Life Technology Co., Ltd., Taipei, Taiwan). At the end of the test, they were asked to immediately stop and sit on a chair for another six minutes; heart rate was repeatedly measured at the end of the test, three minutes, and six minutes after the test. The total distance in meters was recorded and specifically used as the outcome of cardiorespiratory fitness.

### 2.4. Statistical Analysis

Descriptive statistics were conducted using SPSS for Mac ver.26 (IBM Corp., Armonk, NY, USA). All variables were presented in either mean ± standard deviation (SD) or N (%). The Chi-square test was performed to investigate the differences in sex distribution between the DCD and TD groups. In addition, the Mann–Whitney U test was utilized to examine the differences in age, physical fitness, including body composition, flexibility, strength of the lower body, and cardiorespiratory fitness, and PA between the DCD and TD groups. Associations between physical fitness variables and DCD status were evaluated using Spearman’s rank-order correlation coefficients, while relationships between internal factors and PA were examined using Pearson product–moment correlation analysis. Effect sizes for correlation analyses were interpreted based on Cohen’s criteria, with correlation coefficients (r) of 0.10, 0.30, and 0.50 representing small, medium, and large effects, respectively [35,36].

**Figure 1:**
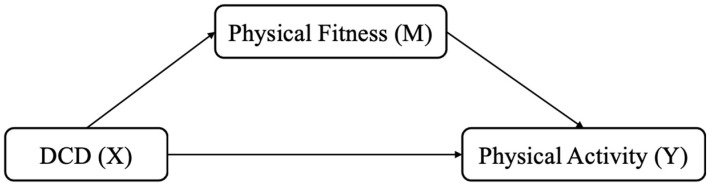
A conceptual diagram of a mediation model tested in this study.

The parallel mediation analysis was conducted using the PROCESS software macro for SPSS version 4.2 (Hayes) [37]. Specifically, we tested whether the association between DCD status (independent variable, X) and physical activity (dependent variable, Y) was indirectly explained through multiple health-related physical fitness indicators (mediators, M), including body mass index, lower-body muscular strength, flexibility, and cardiorespiratory fitness (Figure 1). DCD status was treated as a binary variable (DCD = 1, TD = 0), instead of a continuous variable, in all tested models. It is worth noting that the health-related physical fitness has been chosen as the mediator only if it was significantly correlated with either DCD or PA. Referring to previous studies using mediation analysis [38,39], PROCESS Model 4 was applied, and mediating effects were estimated using bias-corrected 95% confidence intervals derived from 10,000 bootstrap resamples. Bootstrapping was used to estimate indirect effects because such effects are often non-normally distributed; this approach provides robust confidence intervals without relying on normality assumptions, which is particularly appropriate for mediation analysis in smaller samples [37]. A mediating effect was considered statistically significant when the corresponding confidence interval did not include zero. Given the potential influence of age on children’s PA levels [23], chronological age was included as a covariate in all mediation models. In addition, a sensitivity power analysis was conducted using G*Power (version 3.1) for the primary linear multiple regression model underlying the parallel mediation analysis.

## 3. Results

### 3.1. Descriptive Statistics

Descriptive statistics are summarized in Table 1. Compared to the TD group, children with DCD demonstrated significantly worse performance on strength of lower body (*p* < 0.05) and flexibility (*p* < 0.01). Additionally, there were no significant group differences in age, sex, BMI, cardiorespiratory fitness, and PA (all *p*’s > 0.05).

### 3.2. Correlations Between DCD, Internal Correlates, and PA

As shown in Table 2, DCD was significantly and negatively associated with strength of lower body (r = −0.257, *p* < 0.05) and flexibility (r = −0.449, *p* < 0.01). Therefore, based on our definition for potential mediators in this study, only strength of lower body and flexibility were included in the parallel mediation analysis. As there were only four variables (i.e., an independent variable, a dependent variable, and two mediators) in each tested parallel mediation model, our sample size was considered adequate.

### 3.3. Parallel Mediating Effect

**Figure 2:**
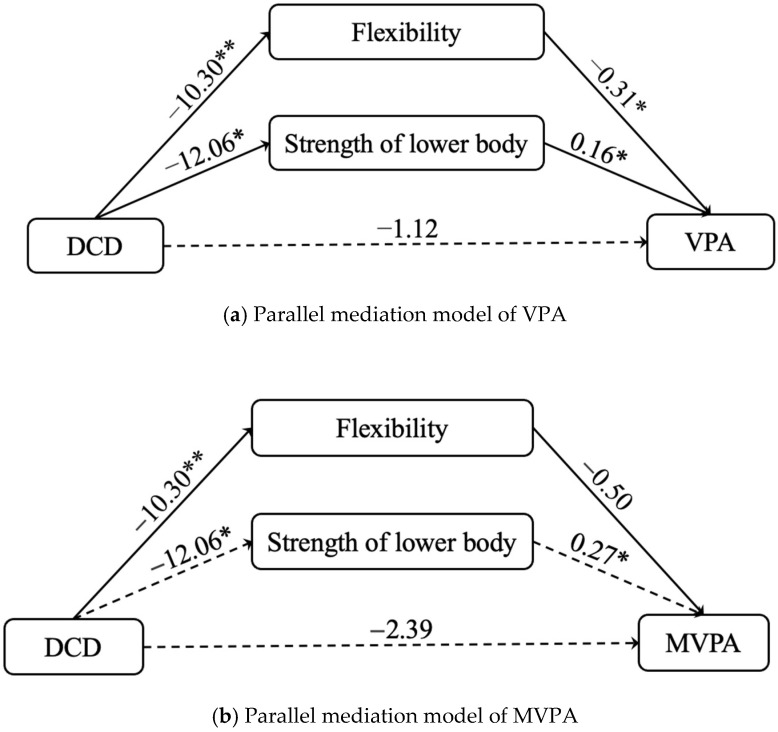

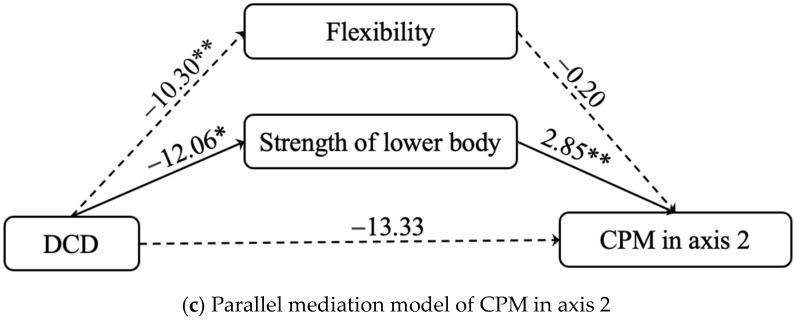
Parallel mediation models with the covariates of age (unstandardized coefficients were presented). * *p* < 0.05, ** *p* < 0.001; MPA, moderate physical activity; VPA, vigorous physical activity; MVPA, moderate-to-vigorous physical activity; CPM: counts per minute, solid lines indicate statistically significant paths (95% bootstrap CI excluding zero), whereas dashed lines indicate non-significant paths.

Parallel mediation models of physical fitness were examined for various PA outcomes, including SB, LPA, MPA, VPA, MVPA, TPA, and CPM in axes 1, 2, and 3, respectively (Table 3). Among these nine models, a significant mediating effect of flexibility was found on the relationship between DCD and VPA (effect = 3.202, bootstrap SE = 1.682, 95% bootstrap CI = 0.463, 7.078; Figure 2a), and DCD and MVPA (effect = 5.194, bootstrap SE = 2.903, 95% bootstrap CI = 0.434, 11.778; Figure 2b). Furthermore, there was a significant mediating effect of strength of lower body on the relationships between DCD and VPA (effect = −1.943, bootstrap SE = 1.297, 95% bootstrap CI = −5.112, −0.021; Figure 2a), DCD and CPM in axis 2 (effect = −34.388, bootstrap SE = 20.212, 95% bootstrap CI = −80.819, −1.353; Figure 2c).

For the sensitivity power analysis, with N = 63, four predictors (PA regressed on DCD status, lower-body strength, flexibility, and age), α = 0.05, and 80% power, the minimum detectable effect size was f^2^ = 0.206 (equivalent to R^2^ ≈ 0.170), indicating adequate sensitivity for detecting moderate-to-large effects, but limited sensitivity for detecting small effects.

## 4. Discussion

To the best of our knowledge, this is the first study to simultaneously examine the potential mediating effects of physical fitness on the relationship between DCD and PA in school-aged children. Our findings lend partial support to our hypotheses, indicating that flexibility may mediate the association between DCD and VPA and MVPA. Moreover, the relationship between DCD and VPA, and DCD and CPM in axis 2, was statistically mediated by strength of lower body. While a limited number of studies are available to support our results, potential interpretations for each significant mediating effect are provided below.

In alignment with previous studies reporting reduced flexibility and poorer muscle strength in children with motor difficulties [20,40,41], our results preliminarily suggest a potential mediating pathway between DCD and PA with higher intensity (i.e., VPA and MVPA) through flexibility. It is worth noting that significant indirect effects may be observed even in the absence of statistically significant differences in PA between the DCD and TD groups. Although conventional mediation approaches often emphasize the presence of a total effect, contemporary perspectives suggest that indirect effects can be meaningfully interpreted even when the total effect is not statistically significant [37]. However, results from the analysis were not fully consistent with our assumptions. It was hypothesized that children with DCD would exhibit poor flexibility, which subsequently would be associated with less engagement in PA. Surprisingly, we found that, although DCD was associated with reduced flexibility, poorer flexibility was associated with increased participation in higher-intensity PA. Given that this finding is counterintuitive, the following interpretations should be considered exploratory rather than conclusive. One possible explanation, which was not directly examined in the present study, is that higher maternal educational attainment may be associated with greater awareness of developmental risks and increased support for children with special needs [42]. Therefore, parents of children with DCD may encourage their children with poor flexibility to participate in PA as a strategy for health promotion. Conversely, TD children with better flexibility may engage in more sedentary lifestyles, such as watching television or playing video games, which could result in less time spent in PA. Additionally, a study has revealed that 8- to 11-year-old Spanish children had limited time for spontaneous leisure activities due to academic demands and structured schedules [43], which may also restrict PA participation. Furthermore, children’s PA levels could also be influenced by their living environment, which is related to the accessibility of safe play spaces, parks, or recreational facilities [44]. However, these factors were not directly assessed in the present study. Therefore, these explanations remain speculative and reflect sample-specific effects, measurement-related factors, or residual confounding. Hence, in addition to internal factors investigated in this study, the association between DCD and PA level is likely to be influenced by a range of external factors, including parental education, parental support, and the environment [45]. Taken together, these findings should be interpreted with caution given the exploratory nature of the mediation analyses and the relatively small DCD sample, and further research is essential to explore the complexities of these interactions on PA in children with DCD.

Another noteworthy finding of this study is the significant mediating effect of strength of lower body on the association between DCD and VPA, suggesting that reduced muscle strength of lower extremities may partially account for why children with DCD are less likely to engage in VPA. Activities with rapid directional changes, such as running and/or jumping during basketball or soccer games, usually require well-developed strength in the lower extremities to be performed effectively and safely [16]. Unfortunately, children with DCD often exhibit decreased neuromuscular efficiency, poor postural control, and lower isometric and dynamic strength, all of which might negatively impact their motor performance during high-intensity tasks [20,46]. Empirical evidence suggests that children with stronger lower-body strength demonstrate higher engagement in VPA, likely due to enhanced movement competence, reduced fatigue, and increased physical self-efficacy [47]. In contrast, children with DCD may avoid or withdraw from vigorous activities because of perceived or actual physical limitations, increased effort required for task execution, or negative experiences associated with physical exertion [48]. These avoidance patterns may contribute to a cycle of physical deconditioning, strength deficits, and limited participation in health-enhancing PA [16]. Accordingly, interventions targeting lower-limb strength may warrant further investigation as a potential approach to help interrupt this cycle and promote greater engagement in VPA in children with DCD. However, given the cross-sectional design and exploratory mediation analyses, these findings should be interpreted cautiously, and causal relationships cannot be established.

While existing research primarily focuses on data retrieved from the vertical axis (axis 1) of the accelerometer to calculate PA intensity, our study examined PA levels across all three axes, as recommended by a recent systematic review [9]. This approach revealed a novel finding, suggesting a potential mediating pathway between DCD and CPM in axis 2 (i.e., horizontal/forward-backward axis) through strength of lower body. Specifically, DCD was associated with lower body strength, which was associated with decreased CPM in axis 2. Although previous evidence has shown an association between motor coordination and strength of lower body [40], the role of lower-body strength in PA, especially CPM in axis 2, remains underexplored. One possible explanation is that certain physical activities, such as free play (e.g., playing with a hoop or climbing a ladder) and soccer-related movements (e.g., dribbling around cones or shooting), may generate higher activity counts in the longitudinal and lateral axes (i.e., axes 2 and 3, respectively) compared to the vertical axis (i.e., axis 1) [49]. These activities often require greater lower limb force production to perform effectively [16,21], which may help explain the observed association between strength of the lower body and CPM in axis 2. Furthermore, consistent with a previous study showing that preschool children aged 3 to 5 years exhibited higher average counts in the longitudinal and lateral axes (axes 2 and 3) [50], our study extends these findings to children aged 6.5 to 8 years. Therefore, we suggest that future studies should consider all three axes rather than focusing solely on the vertical axis. However, given the novelty of this finding and the limited existing evidence, the results should be interpreted with caution. Further research is needed to better understand these relationships and to clarify the potential mechanisms regarding the association between lower-body strength and multi-axis physical activity in children with DCD.

## 5. Limitations

Although our findings provide preliminary insights into the potential roles of flexibility and strength of the lower body on PA among school-aged children with DCD, several limitations should be acknowledged. First, there was an unequal sample size between the DCD and TD groups, which may have limited our ability to detect group differences and warrants cautious interpretation of all results, including between-group comparisons. Second, our approach of screening potential mediators based on observed correlations may increase the risk of selection bias and sample-specific findings and may give the impression of data-driven model specification rather than testing a fully a priori theoretical model. Future research should prioritize theoretically driven model specification to further minimize data-driven findings. Accordingly, the observed indirect effects should be considered preliminary and interpreted cautiously, particularly given the possibility of sample-specific findings. In addition, because multiple fitness and PA outcomes were examined as part of the study objectives, some statistically significant findings may reflect chance variation and should therefore be interpreted cautiously. Moreover, the relatively small DCD sample reduced statistical sensitivity, particularly for detecting small effects. Sensitivity power analysis indicated that the study was primarily powered to detect moderate-to-large effects; therefore, smaller associations may have gone undetected, and the mediation findings should be interpreted as preliminary, pending replication in larger samples. Furthermore, the observed mediation effects were driven primarily by VPA, with no significant mediation observed for MPA, suggesting that the underlying mechanisms linking DCD, physical fitness, and PA may be intensity specific. Finally, future longitudinal or cross-lagged studies are needed to provide more robust evidence regarding changes over time in internal factors and physical activity in children with DCD, as well as to clarify the directionality and causal relationships among these factors. In addition, because physical fitness may interact with psychological characteristics (e.g., stress tolerance and self-efficacy) and contextual factors (e.g., parental behaviors and attitudes, socioeconomic status, and the physical environment) to influence physical activity participation in children with DCD, future research should examine the mediating or moderating roles of these factors to further elucidate the underlying mechanisms.

## 6. Conclusions

Our findings suggest the potential importance of flexibility and muscle strength of the lower extremities in the relationship between DCD and higher intensity PA. Although the findings should be interpreted cautiously, given the exploratory nature of the study, they offer potentially meaningful practical implications for health professionals working with children with DCD. Specifically, when the goal of an intervention is to improve participation in PA among school-aged children with DCD, practitioners may consider incorporating strengthening activities of the lower extremities alongside motor skills training. Furthermore, our findings may suggest that poorer flexibility could be associated with increased engagement in higher intensity PA. This observation may help inform strategies to raise parental awareness regarding the importance of PA; however, this interpretation remains uncertain and requires further investigation. Overall, the findings partially supported the study hypotheses, indicating that significant mediating effects were observed for specific components of physical fitness (i.e., flexibility and muscle strength) on the relationship between DCD and physical activity with higher intensity. However, longitudinal studies are warranted to further clarify these causal relationships.

## Figures and Tables

**Table 1:** Descriptive statistics in children with and without DCD.

	DCD (*n* = 12)	TD (*n* = 51)	*p* Value
Boy (*n*, %)	9 (75.0%)	24 (47.1%)	n.s.
Age (year)	7.89 ± 0.52	7.82 ± 0.62	n.s.
Physical fitness			
BMI (kg/m^2^)	16.82 ± 8.18	16.33 ± 5.03	n.s.
Flexibility (cm)	20.58 ± 8.81	30.95 ± 6.46	**<0.001**
Strength of lower body (cm)	97.50 ± 21.61	108.92 ± 16.89	**0.043**
Cardiorespiratory fitness	501.10 ± 64.93	502.21 ± 90.57	n.s.
Physical activity			
Time			
SB (min/day)	587.00 ± 94.00	581.00 ± 59.00	n.s.
LPA (min/day)	153.00 ± 41.00	158.00 ± 30.00	n.s.
MPA (min/day)	25.00 ± 8.00	26.00 ± 8.00	n.s.
VPA (min/day)	20.00 ± 9.00	20.00 ± 9.00	n.s.
MVPA (min/day)	45.00 ± 16.00	46.00 ± 16.00	n.s.
CPM			
Axis 1	21.00 ± 8.00	22.00 ± 6.00	n.s.
Axis 2	28.00 ± 8.00	30.00 ± 7.00	n.s.
Axis 3	35.00 ± 12.00	34.00 ± 9.00	n.s.

n.s.: no statistical significance. Bold values indicate statistically significant between-group differences. MABC-2: Movement Assessment Battery for Children—Second edition; SB: sedentary behavior; LPA: light PA; MPA: moderate PA; VPA: vigorous PA; MVPA: moderate-to-vigorous PA; CPM: counts per minute.

**Table 2:** Correlations between motor coordination, physical fitness, and physical activity.

		BMI	Flexibility	Strength of Lower Body	Cardiorespiratory Fitness
DCD		−0.140	−0.449 **	−0.257 *	−0.016
SB		0.163	−0.076	0.032	0.182
LPA		0.008	0.150	−0.048	−0.165
MPA		0.013	−0.040	0.057	0.017
VPA		−0.009	−0.098	0.114	0.058
MVPA		0.003	−0.073	0.090	0.044
TPA		0.008	0.086	−0.003	−0.107
CPM	Axis 1	−0.058	−0.022	0.067	−0.015
	Axis 2	−0.161	0.206	0.234	−0.103
	Axis 3	−0.024	0.206	−0.023	−0.098

* *p* < 0.05; ** *p* < 0.01. Bold values indicate statistical correlations between variables. DCD: developmental coordination disorder; SB: sedentary behavior; LPA: light PA; MPA: moderate PA; VPA: vigorous PA; MVPA: moderate-to-vigorous PA; TPA: total PA; CPM: counts per minute.

**Table 3:** The parallel mediating effects of physical fitness on the relationship between motor coordination and physical activity.

		Flexibility				Strength of Lower Body			
		Effect	Bootstrap SE	95% Bootstrap Confidence Interval		Effect	Bootstrap SE	95% Bootstrap Confidence Interval	
				Lower Limit	Upper Limit			Lower Limit	Upper Limit
SB		0.306	12.162	−24.192	23.977	4.353	8.337	−8.713	24.894
LPA		−3.944	5.041	−14.108	6.180	−1.243	2.750	−8.096	3.346
MPA		2.063	1.334	−0.187	5.020	−1.287	0.944	−3.562	0.074
VPA		**3.202**	**1.682**	**0.463**	**7.078**	**−1.943**	**1.297**	**−5.112**	**−0.021**
MVPA		**5.194**	**2.903**	**0.434**	**11.778**	−3.208	2.201	−8.526	0.053
TPA		1.193	6.705	−11.419	15.404	−4.432	4.380	−15.551	1.501
CPM	Axis 1	27.533	27.140	−10.995	76.523	−23.091	17.466	−66.262	0.588
	Axis 2	2.027	23.598	−42.659	51.589	**−34.388**	**20.212**	**−80.819**	**−1.353**
	Axis 3	5.602	29.767	−52.542	67.104	−20.285	21.400	−75.029	6.105

Note. Bold indicates a significant mediating effect. SB: sedentary behavior; LPA: light PA; MPA: moderate PA; VPA: vigorous PA; MVPA: moderate-to-vigorous PA; TPA: total PA; CPM: counts per minute.

## Data Availability

The datasets generated and/or analyzed during the current study are not publicly available due to ethical and privacy considerations related to research involving children but are available from the corresponding authors upon reasonable request.

## Abbreviations

The following abbreviations are used in this manuscript:

DCD	Developmental coordination disorder
TD	Typically developing
DSM-V	Diagnostic and Statistical Manual of Mental Disorders, Fifth Edition
PA	Physical activity
SB	Sedentary behaviour
LPA	Light physical activity
MPA	Moderate physical activity
VPA	Vigorous physical activity
MVPA	Moderate to vigorous physical activity
TPA	Total physical activity
CPM	Counts per minutes
